# An Examination of Gross Lesions Associated with Bovine Tuberculosis in the U.S.

**DOI:** 10.3390/pathogens15040350

**Published:** 2026-03-26

**Authors:** Cara C. Drehoff, Kent C. Munden, Christa Ray, Heather Martinez, Suelee Robbe-Austerman, Jason E. Lombard

**Affiliations:** 1 Oak Ridge Institute for Science and Education, Oak Ridge, TN 37830, USA; 2 Field Epidemiologic Investigation Services, Veterinary Services, Animal and Plant Health Inspection Service, United States Department of Agriculture, Fort Collins, CO 80526, USA; 3 Field Operations, Veterinary Services, Animal and Plant Health Inspection Service, United States Department of Agriculture, Austin, TX 78701, USAchrista.l.ray@usda.gov (C.R.); 4 National Veterinary Services Laboratories, Veterinary Services, Animal and Plant Health Inspection Service, United States Department of Agriculture, Ames, IA 50010, USA

**Keywords:** bovine TB, lesions, diagnostic tests

## Abstract

Understanding the gross lesion distribution associated with bovine tuberculosis (bTB) and the relationship between antemortem test results is important for optimizing surveillance activities. Antemortem diagnostic test results and postmortem examination results from animals infected with bTB in the United States are routinely collected during surveillance and outbreak response. From 2017 to 2022, data were compiled and analyzed from 403 infected animals, representing both beef and dairy cattle from a variety of herds with different levels of disease prevalence. Overall, 95.3% of cattle infected with *Mycobacterium bovis* presented with gross lesions. Most cattle had lesions in one anatomic region. Lesions of the thorax and head were most common with 42.4% of infected cattle having lesions in only the thorax, 18.4% having lesions only in the head, and 15.6% having lesions in both the head and thorax. The most frequently affected tissues were the mediastinal, tracheobronchial, and medial retropharyngeal lymph nodes. Results of antemortem skin tests were not found to be associated with lesion count or location. This analysis presents an updated look at the current state and presentation of bTB in the U.S., makes use of data collected in the field, and can help guide future bTB surveillance and control strategies.

## 1. Introduction

The causative agent of bovine tuberculosis is the acid-fast bacillus *Mycobacterium bovis* [1]. *M. bovis*, a member of the *Mycobacterium tuberculosis* complex, has a broad host range infecting numerous mammalian species from domesticated animals and wildlife to humans, in which it is known as zoonotic tuberculosis [2]. In the early 1900s, bovine tuberculosis (bTB) was a major threat to the U.S. livestock industry and a significant public health issue mainly due to the consumption of raw dairy products from tuberculous cows [1]. In response, the U.S. Department of Agriculture (USDA) created the National Tuberculosis Eradication Program in 1917. The program, a collaboration between federal and state governments, has since been very successful, lowering the cattle-level prevalence of bTB from approximately 5% in 1917 to <0.000008% in 2023 [3]. Despite such a low prevalence in cattle herds, this disease continues to have economic, animal health, and public health implications.

Cattle are commonly infected with *M. bovis* via inhalation or ingestion, with calves most commonly exposed via milk [4,5]. The disease name is derived from the characteristic formation of tubercles, or granulomas, which are a result of the host’s cell-mediated immune response [1,4]. Granuloma formation by the host is a mechanism to contain and eliminate the pathogen by limiting mycobacterial proliferation [6]. The location and extent of these lesions are determined by several factors, including exposure route and dose, host immune response, and pathogen characteristics [4,7].

As bTB prevalence in U.S. cattle is low and clinical signs from advanced disease are rarely seen, the USDA’s National Tuberculosis Eradication Program relies on the detection and submission of gross lesions at slaughter as the primary method for bTB surveillance [8]. Antemortem diagnostic tests, including skin and blood tests, are also used in both surveillance and disease control. All tests involve stimulation with *M. bovis* antigen and measure subsequent host cell-mediated immunity. Because these tests rely on host immune response rather than pathogen detection, results may be affected by the state of disease progression or latency [9]. The caudal fold tuberculin (CFT) skin test is used for screening, and if positive, is followed by one or both of the official, secondary confirmatory tests, the comparative cervical tuberculin (CCT) skin test and/or the gamma interferon blood test. These tests are used to confirm disease, and if either or both are positive, indicate a need for postmortem testing.

After a herd is determined to be infected with bTB, animals suspected to be infected based on antemortem testing are euthanized to facilitate quick identification and elimination of bTB. The carcasses of these animals are inspected for gross lesions, and samples are submitted to the USDA’s National Veterinary Services Laboratories (NVSL) for confirmatory testing. As the prevalence of bTB continues to decline in U.S. cattle, detection of this pathogen will become increasingly challenging. While experimental studies are incredibly valuable in understanding pathogen characteristics and disease pathogenesis, studies of natural infection may be more relevant for disease detection and management. Studies evaluating data originating from naturally infected herds in the U.S. have been scarce. The objectives of this comparative epidemiological study were to: (1) describe the location of bTB gross lesions from naturally infected cattle, and (2) evaluate the association between bTB gross lesion location and ante-mortem test results.

## 2. Materials and Methods

Herds selected for inclusion in this retrospective analysis were detected within the last 10 years and completed depopulation or test-and-removal procedures to eliminate bTB. All herds included not only had sufficient reporting of gross lesion data and laboratory results but also underwent epidemiologic investigation into disease source and spread. Data for this study were gathered from multiple USDA and state-level reports and databases. Gross lesion data were obtained from USDA-Animal and Plant Health Inspection Service-Veterinary Services forms 6-35 and 10-7, which report granulomas found at slaughter and lesions found at necropsy of cattle from a bTB-infected herd, respectively. Paper forms were submitted with tissue samples and scanned upon arrival to the NVSL. The digital copies of these forms were transcribed into a Microsoft Excel spreadsheet and imported into SAS 9.4 (SAS Institute Inc., Cary, NC, USA) for data analysis. Animal age and antemortem testing results were also transcribed from these forms. When available, animal age was verified using the date of birth from herd owner records. Additionally, antemortem testing results were verified using federal or state testing records when available. Definitive diagnoses for each animal were obtained via a query of the NVSL STRAND (Searchable Test Results Application for NVSL Diagnostics) database. An animal was classified as infected with bTB if postmortem lab results showed a positive bacteriologic culture or, in the case of a negative culture, the pathogen was detected on both polymerase chain reaction and histopathology analysis.

Gross lesion reporting was variable, with some examiners reporting the specific lesioned tissue and others reporting only the regional location of the lesion. Thus, this analysis focuses on the region of lesion location and discusses specific sites of lesions only where available. Analyses of lesion count and lesion location were conducted on an individual animal basis. Lesion count was defined as the number of distinct specific tissues affected, not the number of lesions within one tissue, as that information was not collected.

Descriptive and statistical analyses were completed using SAS. Statistical tests conducted involving continuous variables included the one-way ANOVA test and the *t*-test. For associations between categorical variables, the chi-squared test, or Fisher’s exact test when greater than 20% of cell frequencies were less than 5, was used. Figures were created in Microsoft Excel and Adobe Photoshop.

## 3. Results

**Figure 1:**
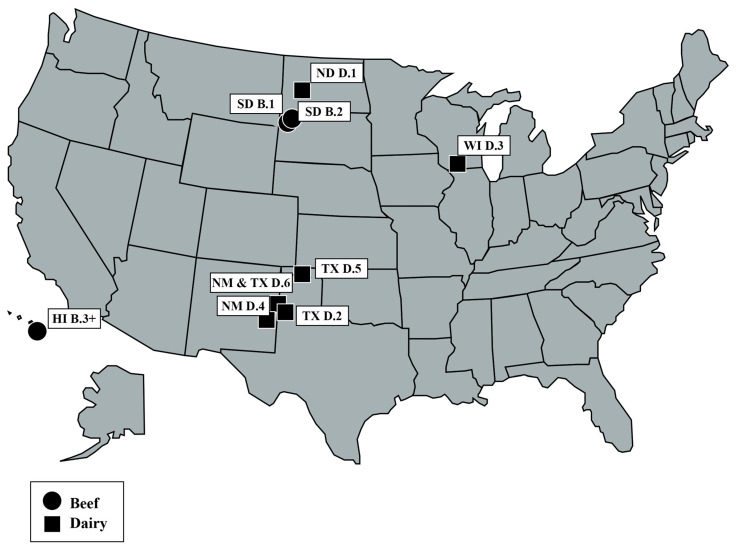
U.S. map of nine bovine tuberculosis-infected herds mapped to the center of their county location.

Data for analysis were compiled from nine infected U.S. herds, which represent both beef (n = 3) and dairy (n = 6) cattle as well as a variety of geographic locations, herd sizes, sources of introduction, and levels of intra-herd disease prevalence (0.1–16.3%). Details on these herds can be found in Table 1 below. The location of the herds presented is mapped to the center of their county in Figure 1 below.

### 3.1. Beef vs. Dairy Cattle

**Figure 2:**
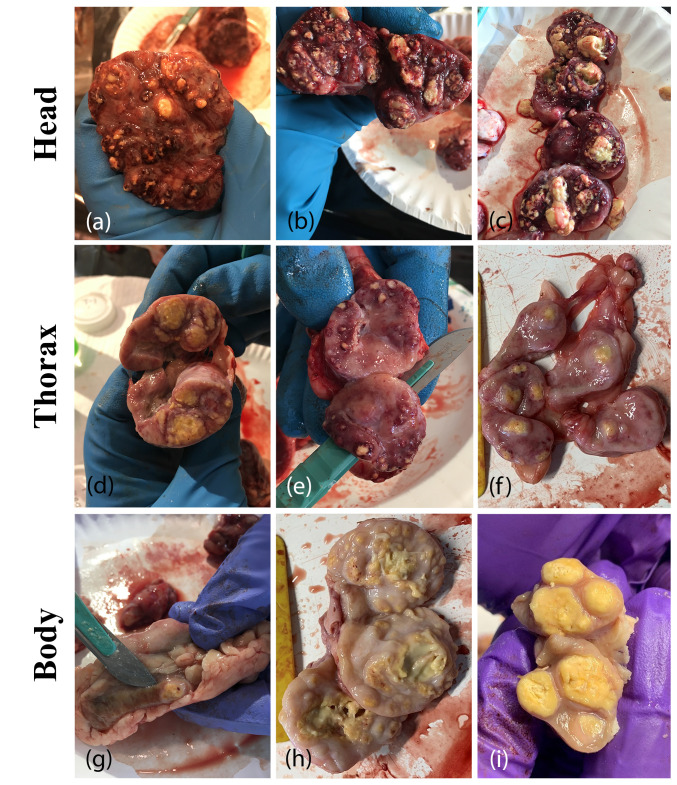
Typical bovine tuberculosis lesions detected in the head (**a**–**c**), thorax (**d**–**f**), and body (**g**–**i**), from infected cattle in Herd D.5 (Photos courtesy of Dr. Christa Ray).

**Figure 3:**
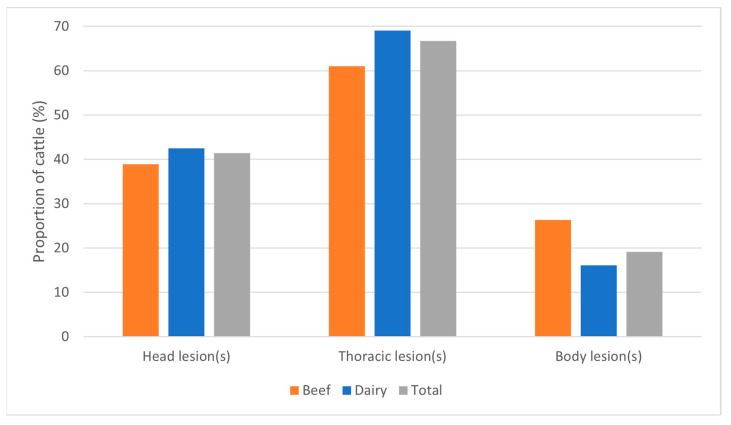
Lesion distribution in bovine tuberculosis-infected cattle by production type. Lesion categories are not exclusive, i.e., the same cow may be represented in multiple categories if lesions were present in more than one region.

The average age of infected cattle was 46.6 months of age, with beef cattle being significantly older on average than dairy cattle (Table 2). One possible reason for the age differences may be attributable to the longevity of beef cattle compared with dairy cattle. The mean age and distribution of beef cattle are older and broader, respectively, compared with dairy cattle. This may be due in part to higher cull rates in dairy cattle when compared to beef cattle [10,11]. Modeling results suggest that bTB detection in small- and medium-sized beef herds takes longer than in dairy herds because of the smaller percentage of the adult herd that is removed to slaughter and inspected each year [12]. This delay in detection could lead to more within-herd spread and the beef herds in this analysis did have higher herd-level prevalence than dairy herds. Overall, most cattle infected with bTB presented with gross lesions (95.3%) with no difference between beef and dairy. Most often, these cattle had lesions in only one body region, with the majority having lesions only in the thorax, followed by only in the head. Selected lesion photos from Herd D.5 are presented in Figure 2. Beef and dairy cattle showed similar trends in lesion count and lesion location (Figure 3). Although a greater number of beef cattle presented with multiple lesions (41.5% vs 30.9%), this finding was not statistically significant. A higher percentage of beef than dairy cattle had any lesions in the body region (26.3% vs 16.1%, *p* = 0.019 and only lesions in the body region (11.9% vs 3.9%, *p* = 0.002).

### 3.2. Specific Sites of Gross Lesions in bTB-Infected Cattle

**Figure 4:**
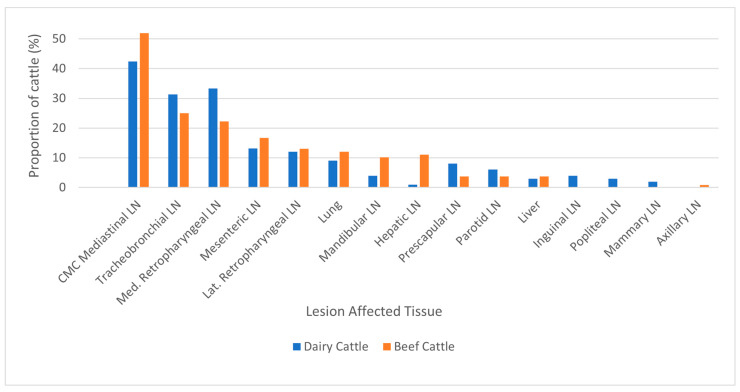
Lesioned tissues in lymph nodes (LN) and organs from bovine tuberculosis-Infected cattle by production type. 99/272 infected dairy cattle and 108/112 infected beef cattle with lesions had the specific organ of lesion location reported, whereas the remainder had only the region of lesion location(s) (head, thorax, body) and thus were excluded. CMC = Cranial, Middle, Caudal.

When available, data on the specific tissue affected with gross lesions in bTB-infected animals were analyzed. Overall, the mediastinal lymph nodes were most affected, followed by the tracheobronchial and medial retropharyngeal lymph nodes, being lesioned in 47.3%, 28.0% and 27.5% of all cattle, respectively (Table 3 and Figure 4). Beef and dairy cattle showed similar patterns in lesion distribution. Notably, beef cattle were significantly more likely to have lesions in the hepatic lymph node (11.1% and 1.0%, respectively).

### 3.3. Herd Stratification

Gross lesion presentation differed among the herds included in this study. Most infected herds had at least 77% of the cattle with gross lesions (Table 4). Notably, no gross lesions were found in the three infected cattle from D.1. While most herds predominantly had animals with a single lesion, multiple lesions were observed in at least half of the animals from herds D.3 and D.6. With regard to regional lesion distribution, patterns were variable between herds. In five of the herds, thoracic lesions alone were the most frequent pattern observed. However, in herd D.4, head lesions alone were found more often, and in herd D.3, body lesions predominated both on their own and in conjunction with other regions.

### 3.4. Antemortem Skin Testing

**Figure 5:**
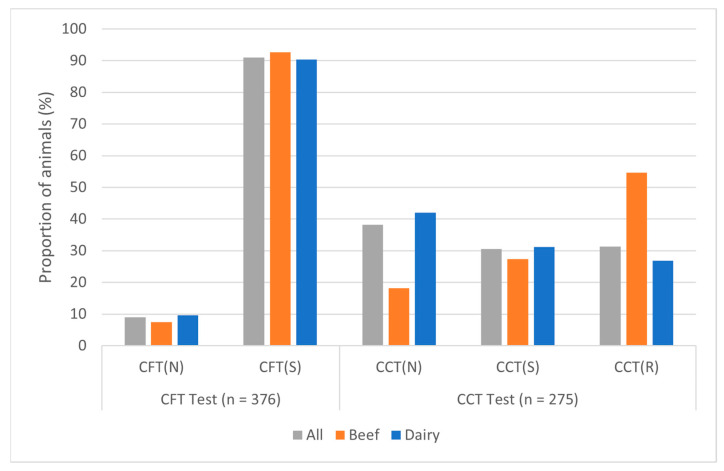
Antemortem skin-test results for bovine tuberculosis-infected cattle by breed. CFT = caudal fold tuberculin, CCT = comparative cervical tuberculin, (N) = negative, (S) = suspect, (R) = reactor.

Antemortem test results presented in Table 5 were based on the most recent test/series of tests conducted and were therefore those most proximate to the animal’s death. Not all infected animals received any and/or all tests due to the method of detection (slaughter vs herd removal), detection in trace-outs, and differing antemortem testing strategies. The CFT test showed high levels of sensitivity (>90%) and similar performance in both beef and dairy cattle. This sensitivity estimate should be interpreted with caution since cattle with negative CFT results were not generally examined by necropsy. Although many CFT-negative cows from these herds went through slaughter surveillance, this method of detection is not as sensitive as necropsy. The CCT test, however, performed significantly different (*p* = 0.003) between beef and dairy cattle, with the CCT positivity rate, defined as those classified as either suspect or reactors, being 81.8% in beef compared to only 58.0% in dairy cattle. Test performance stratified by herd was variable. Although CFT performance was relatively consistent among herds and similar if not greater than established sensitivity levels [13], CCT results were more variable between breeds (Figure 5).

**Figure 6:**
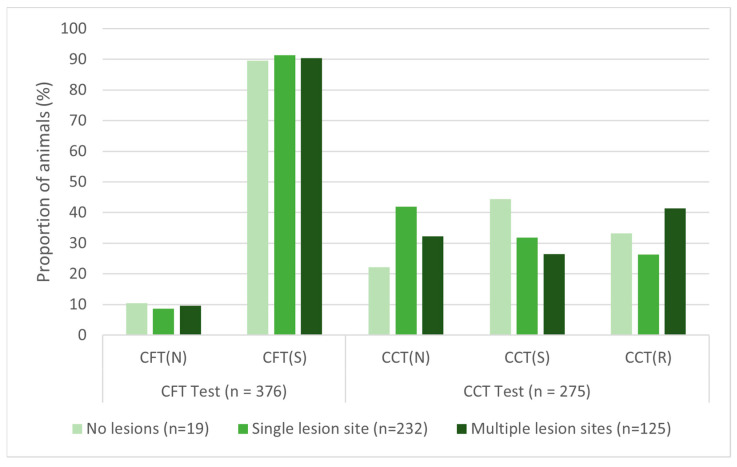
Antemortem skin-test results for bovine tuberculosis-infected cattle by lesion count. CFT = caudal fold tuberculin, CCT = comparative cervical tuberculin, (N) = negative, (S) = suspect, (R) = reactor.

There were no significant associations found between antemortem test results and lesion count for either skin test (Table 6). The sensitivity of the CFT test was within 2% among animals with zero, one, or multiple lesion sites present, while CCT sensitivity was more variable (Figure 6). Additionally, there were no associations between skin test results and lesion presence in specific body sites, except that cattle with lesions only in the body region were significantly less likely to show a response to CFT testing than all other lesion location cohorts.

## 4. Discussion

### 4.1. Overall Gross Lesion Findings

The sensitivity of gross lesions for the detection of bTB-infected cattle was 95.3%, which was higher than reported from a study evaluating seven herds in Michigan, USA, where the sensitivity of gross lesions was 86.0% [14]. The presence and extent of gross lesions from bTB are known to vary with route of exposure, pathogen dose and virulence, time between infection and diagnosis, and host immune response [7]. The predominance of lesions in the thoracic region is consistent with findings from experimental and natural infection with *M. bovis* in previous studies [6,15,16]. It is widely accepted that the method of exposure plays a large role in the location and distribution of gross lesions in bTB infection [7,17]. Therefore, it is reasonable to speculate that the most common source of transmission to cattle included in this analysis was respiratory. In experimental studies exposing cattle to *M. bovis* via aerosol delivery, gross lesions were most often found in the lungs and associated lymph nodes [18,19,20]. In this analysis, lung lesions were only noted in 10.6% of cattle, which may indicate a need for improvement in postmortem lung inspection.

In this analysis, 15% of cattle presented with lesions in the mesenteric Ln. In other studies of gross lesions resulting from natural infection with *M. bovis*, the mesenteric Ln. represented 0–10% of lesions [15,16,21]. However, a study from China reported more than 90% of lesions in 151 necropsied cattle were extrapulmonary; more than 70% of lesions were in the gastrointestinal tract, and 43% were in the mesenteric Ln. [22]. Mesenteric Ln., and others in or adjacent to the gastrointestinal tract, may be affected from exposure to *M. bovis* via ingestion [1,4]. This method of exposure is generally thought to be most relevant for calves who may consume *M. bovis* from contaminated milk or be in close contact with their dams [4,22,23]. Based on this analysis, ingestion may be a more common transmission route in the U.S. than previously thought, occurring in older cohorts of cattle as well as calves. Alternatively, lymph nodes and other organs not associated with the respiratory tract may be affected with gross lesions from disseminated infection. This would likely occur later in the disease process via hematogenous or lymphatic spread, or perhaps secondary to the swallowing of contaminated sputum [7,17]. However, of those animals with lesions in the mesenteric lymph nodes (n = 31), 35.5% presented with lesions in the body region alone, indicating that the region may have been the primary site of infection. As we cannot determine the chronological development of these lesions, it is impossible to differentiate respiratory exposure with dissemination to the abdomen from alimentary exposure with dissemination to the thorax.

Of note is that 4.7% of infected animals in this study did not have any gross lesions, and this percentage is likely an underestimate of the infected cattle population. This finding illustrates the importance of testing samples from animals suspected to have bTB, regardless of the presence of gross lesions.

### 4.2. Beef vs. Dairy

Beef and dairy cattle were compared as these groups involve different breeds and management practices. As noted in Table 1, the prevalence of disease in beef herds was higher on average than in dairy herds. There are several reasons why the detection of *M. bovis* in beef herds may be more challenging, thereby allowing the disease to spread for a greater duration than in dairy herds. The beef industry has a much lower culling (permanent removal) rate than the dairy industry—approximately 13% vs. 30%—meaning that the chance of finding an infected herd via an animal at slaughter is decreased due to sample size alone [10,11]. Compounding this is the fact that beef herds, as shown in this analysis, tend to be smaller than dairy herds. The prevalence in beef herds in this study may be exaggerated, as all beef herds were selected for whole-herd depopulation. Therefore, each beef animal underwent either a slaughter or necropsy inspection, which could have resulted in the detection of additional infected animals that may not have been detected by antemortem testing or slaughter surveillance alone. Alternatively, or in conjunction, the management of beef herds may make cow-to-cow transmission more likely, as beef herds tend to comingle animals of different ages, thereby potentially exposing young, vulnerable animals to infected older ones, although this is a risk at some dairies too.

Beef animals were significantly more likely to present with lesions in the body only (11.9% vs. 3.9%, *p* = 0.002). This suggests beef cattle are exposed to the pathogen more often via ingestion than dairy cattle. Interestingly, beef cattle had a significantly higher proportion of lesions in the hepatic lymph node than dairy cattle, which could be attributed to the filtration of contaminated fluids from the gastrointestinal tract. However, a difference was not found at other related sites such as the liver or mesenteric ln. It is also worth noting that there was a significant difference in the age of infected cattle, with the average age of beef cattle being approximately two years greater than that of dairy cattle, which could have had an impact on lesion development and presentation as disease progresses over time.

### 4.3. Variation Between Herds

As this analysis includes animals from nine different herds across the country exposed to *M. bovis* via natural infection, there were many factors that could account for variation in disease development or presentation among herds. The epidemiologic triad of host, pathogen, and environment provides a framework for these potential factors. Host-related factors include immune status at the time of exposure and duration of illness. Herd-related characteristics could include genetics, nutrition, other comorbidities, and management practices. Environmental factors include stocking density and ventilation, cleanliness, and climate. Pathogen-related factors include the virulence of different strains, the portal of entry, and the dose received. Notably, each herd included in this analysis was predominantly infected by a unique *M. bovis* strain, except for Herd D.5. The majority of the cows had the 17B1 strain. In addition, Herd D.5 had a single cow infected with a Group 6A strain similar to Herd B.2, although the strains were not closely related. Herd D.5 also had a single cow infected with Group 13A. However, some studies conclude that livestock management practices may have a greater impact on disease severity than strain [20]. Future studies should focus on herd-related characteristics to determine the impact of practices on the number and location of bTB lesions.

Although overall trends emerged, there was noticeable variability in lesion count and location, with some herds having unique presentations. In all herds excepting D.1, the majority of cattle presented with gross lesions. Among these eight herds, it was more typical that animals had single lesions, although the proportion with multiple lesions ranged from 27.4 to 58.3%. Variability in the number of lesions between herds may relate to disease chronicity or animal susceptibility. Often, when an infected herd is detected, it is difficult to determine the time since exposure. However, D.1 was confirmed to be infected via human-to-cattle transmission and whole-herd testing was conducted only one month after the human case was detected [24]. The absence of gross lesions in this herd supports that these animals may have been detected very early in the disease process. By contrast, herd D.3, another confirmed human-to-cattle transmission, was detected three years after presumed exposure and the majority of cattle presented with multiple lesions. Epidemiologic investigation and whole genome sequencing also confirmed cow-to-cow transmission in this herd.

With respect to lesion location, five of eight herds with lesions had the highest proportion of animals present with lesions in the thorax only. Uniquely, animals in D.4 were more often affected with lesions in the head only, while herd D.3 had no predominant presentation. Animals from Hawaiian herds were also variable in presentation, with thoracic lesions only and body lesions only being most common and of equal occurrence. There are many factors that could influence lesion location and dissemination, including pathogen dose, route, disease chronicity, and individual animal characteristics. While the definitive causes of variation cannot be identified in this analysis, perhaps the greater takeaway is the variation alone. As animal health officials seek to identify and eliminate bTB from U.S. herds, recognizing bTB in its variable presentations will be essential.

### 4.4. Antemortem Skin Testing

Antemortem tests for bTB are known to be imperfect, with the CFT skin test and CCT skin test having a median sensitivity of approximately 84% and 80%, respectively [13,25]. The overall sensitivity of the CFT test in this cohort of animals was 91%, which is higher than reported but similar to the 93% reported in a Michigan, USA Study [14]. This result is biased as animals with a positive CFT test were more likely to undergo postmortem inspection. The performance of the CCT differed significantly between beef and dairy animals. The CCT had a higher sensitivity in beef than in dairy animals (81.8% vs. 58.0%). It is difficult to explain this variation, but it is worth considering the subjectivity of the tuberculin skin tests as well as the potential role of disease chronicity in beef animals. Additionally, there was a large discrepancy between the number of available CCT results for beef vs. dairy animals (44 vs. 231, respectively), which may impact interpretation. Variability in the performance of these tests between herds was similar. While the CFT was consistent among herds with a sensitivity of approximately 80% or higher, CCT performance varied with a sensitivity from 41.2 to 100 addition, test responses may differ by tuberculin dose and purified protein derivative preparations, animal stress level, or stage of disease [25].

Antemortem tests for bTB do not rely on the detection of the pathogen, but rather on the host’s immune response when stimulated with an antigen. Therefore, it was hypothesized that animals with a greater number of lesions would be more likely to be detected. This assumes that multiple lesion sites indicate more severe disease than a single lesion, as lesion size was not commonly reported. Surprisingly, as seen in Figure 5 above, there was no significant association or consistent trend between skin test results and the number of lesions. Not finding a difference in immune response in cattle with lesions in multiple sites and those with single or no lesions could suggest that the lack of a robust immune response might lead to more disseminated infection. It is worth noting that animals without gross lesions were a very small proportion of the infected cattle, and therefore, these results must be interpreted with caution. Lastly, there were no notable associations between lesion location and skin test result, except that cattle with lesions in only the body region had lower sensitivity on CFT testing. This has also been reported in experimentally infected calves [26]. Alternatively, body lesions may not be detected at slaughter because these tissues are not as carefully examined as the thoracic and head tissues [16].

### 4.5. Limitations

There are several limitations to acknowledge in this study, as the data comes from the field and was not originally collected for the purpose of this analysis. Even though not collected for this retrospective study, the data are routinely collected based on guidance in the Bovine Tuberculosis Eradication Uniform Methods and Rules (UMR) [27]. Official, standardized forms, surveillance guidelines, and testing protocols are outlined in the UMR. Results and conclusions rely on the accurate and thorough inspection and reporting of gross lesions from individuals completing necropsies and slaughter inspections. Data quality and completeness may differ depending on who is completing this task, their skills and knowledge base, and any time constraints they may be working under. For example, slaughter inspection may be less thorough than necropsy due to quick line speeds and logistics regarding the splitting of the carcasses. In addition, a lot of emphasis is placed on head and thoracic evaluation for lesions during the slaughter process. This is because historically, the majority of lesions are in these 2 regions, and because the abdominal organs are removed without the same level of inspection [14]. Second, the consistency of inspection and reporting may differ as the 403 infected animals included in this study were examined by 35 different individuals across the U.S. However, it is worth noting that 208 necropsies were completed by the same person. Third, animals with gross lesions may be overrepresented, as during certain periods, lesioned samples were prioritized for collection and laboratory testing due to human resource and lab capacity constraints. Therefore, as this sample is biased toward cattle with lesions, the percentage of infected cattle without gross lesions may be understated. Finally, lack of granularity was a limitation in this study, as the specific number or size of lesions in each organ was not recorded.

## 5. Conclusions

As the National Tuberculosis Eradication Program continues to strive to eliminate bTB, it is worth contemplating how to modify current approaches. bTB lesions in only the body were found in about 6% of cattle and almost 90% of cattle had bTB lesions in lymph nodes of the head and/or thorax. Current necropsy and slaughter sample collection recommendations for bTB control and surveillance should remain and additional emphasis should be placed on more thorough carcass inspections, including the lungs and body lymph nodes. The finding that beef cattle were more likely to have body lesions than dairy cattle could result in more targeted slaughter surveillance based on breed. Furthermore, the fact that not all infected animals had gross lesions, and would be missed on slaughter surveillance, highlights the importance of histopathology of tissues from test-positive animals regardless of the presence of lesions. In the pursuit of disease eradication, it is essential to use information collected from ongoing and past outbreaks to guide future surveillance and control strategies. Despite over 100 years of targeted disease eradication work, there is still much to learn about bovine tuberculosis. To our knowledge, this paper is the first to examine gross lesion data resulting from natural bTB infection across multiple U.S. cattle herds. This analysis provides further insight into bTB control, including exposure and transmission mechanisms, predilection sites, and antemortem testing patterns.

## Figures and Tables

**Table 1:** Bovine tuberculosis-infected herd characteristics.

Herd ID	Year of Detection	Herd State(s)	Production Type	Breed	Herd Size	Total Infected	Herd Prevalence (%)	*M. bovis* Strain(s)/ Group	Likely Source of Introduction
B.1	2017	South Dakota	Beef	Angus	656	46	7.0%	7B	Unknown
B.2	2017	South Dakota	Beef	Angus	338	63 *	16.3%	6A	Unknown
B.3 †	2021	Hawaii	Beef	Angus, Corriente, Brahman	1100	16	1.5%	5A	Wildlife spillover
D.1	2013	North Dakota	Dairy	Holstein	400	3	0.8%	8F	Human-to-cattle
D.2	2014	Texas	Dairy	Holstein	10,000	158	1.6%	16C	Unknown
D.3	2018	Wisconsin	Dairy	Holstein	1500	12	0.8%	17B3	Human-to-cattle
D.4	2019	New Mexico	Dairy	Holstein	21,000	31	0.2%	24	Unknown
D.5	2019	Texas	Dairy	Holstein	9000	75	0.8%	17B1, 13A, 6A	Unknown
D.6	2022	New Mexico and Texas	Dairy	Holstein	12,000	8	0.1%	22	Unknown

* Including several trace-outs from this herd. † Herd B.3 groups several infected herds due to the small size of the herds.

**Table 2:** Summary of lesions in bovine tuberculosis-infected cattle by production type.

Characteristic (Number of Infected Cattle)	Beef Cattle n = 118	Dairy Cattle n = 285	*p*-Value	Total n = 403
Age at necropsy (months) *, mean ± SD	64.8 ± 29.1	39.1 ± 22.4	**<0.001**	46.6 ± 27.2
Lesion Count			0.104	
No lesion present, n (%)	6 (5.1)	13 (4.6)		19 (4.7)
Any lesion present, n (%)	112 (94.9)	272 (95.4)		384 (95.3)
Single site with lesion present, n (%)	63 (53.4)	184 (64.6)		247 (61.3)
Multiple sites with lesions present, n (%)	49 (41.5)	88 (30.9)		137 (34.0)
Lesion Location(s) **				
Any Head Lesions	46 (38.9)	121 (42.5)	0.520	167 (41.4)
Any Thorax Lesions	72 (61.0)	197 (69.1)	0.116	269 (66.7)
Any Body Lesions	31 (26.3)	46 (16.1)	**0.019**	77 (19.1)
Head Lesion(s) Only	21 (17.8)	53 (18.6)	0.850	74 (18.4)
Thorax Lesion(s) Only	43 (36.4)	127 (44.6)	0.133	170 (42.4)
Body Lesion(s) Only	14 (11.9)	11 (3.9)	**0.002**	25 (6.2)
Head and Thorax Lesions	17 (14.4)	46 (16.1)	0.663	63 (15.6)
Head and Body Lesions	5 (4.2)	11 (3.9)	0.860	16 (4.0)
Thorax and Body Lesions	9 (7.6)	13 (4.6)	0.218	22 (5.5)
Head, Thorax, and Body Lesions	3 (2.5)	11 (3.9)	0.511	14 (3.5)

* Specific information on age is missing for 30 infected cattle: 9 beef and 21 dairy. ** See Appendix A Table A1 for specific tissues included in each region.

**Table 3:** Lesioned tissues in lymph nodes (LN) and organs from bovine tuberculosis-infected cattle by production type *.

Region	Tissue	Beef Cattle (n = 108 *)	Dairy Cattle (n = 99 *)	*p*-Value	Total (n = 207)
Thorax	Cranial, Middle, Caudal Mediastinal LN	56 (51.9)	42 (42.4)	0.175	98 (47.3)
Thorax	Tracheobronchial LN	27 (25.0)	31 (31.3)	0.312	58 (28.0)
Head	Medial Retropharyngeal LN	24 (22.2)	33 (33.3)	0.074	57 (27.5)
Body	Mesenteric LN	18 (16.7)	13 (13.1)	0.477	31 (15.0)
Head	Lateral Retropharyngeal LN	14 (13.0)	12 (12.1)	0.855	26 (12.6)
Thorax	Lung	13 (12.0)	9 (9.1)	0.492	22 (10.6)
Head	Mandibular LN	11 (10.2)	4 (4.0)	0.089	15 (7.3)
Body	Hepatic LN	12 (11.1)	1 (1.0)	0.003	13 (6.3)
Body	Prescapular LN	4 (3.7)	8 (8.1)	0.237	12 (5.8)
Head	Parotid LN	4 (3.7)	6 (6.1)	0.430	10 (4.8)
Body	Liver	4 (3.7)	3 (3.0)	1.0	7 (3.4)
Body	Inguinal LN	0 (0.0)	4 (4.0)	0.051	4 (1.9)
Body	Popliteal LN	0 (0.0)	3 (3.0)	0.108	3 (1.5)
Body	Mammary LN	0 (0.0)	2 (2.0)	0.228	2 (1.0)
Body	Axillary LN	1 (0.9)	0 (0.0)	1.0	1 (0.5)

* 99/272 infected dairy cattle and 108/112 infected beef cattle with lesions had the specific organ of lesion location reported, whereas the remainder had only the region of lesion location(s) (head, thorax, body) and thus were excluded. Cattle will be represented in multiple rows if they have multiple specific lesions reported, thus percentages will not sum to 100%. LN = lymph node.

**Table 4:** Summary of lesion location for bovine tuberculosis-infected cattle by herd.

Herd (Number of Infected Cattle)	B.1 n = 46	B.2 n = 56	B.3 n = 16	D.1 n = 3	D.2 n = 158	D.3 n = 12	D.4 n = 31	D.5 n = 73	D.6 n = 8
Age at necropsy (months) *, mean ± SD	62.3 ± 14.8	74.5 ± 33.3	39.6 ± 30.9	38.3 ± 16.7	27.8 ± 19.1	58.3 ± 11.8	36.7 ± 14.9	57.5 ± 19.1	48.9 ± 4.6
Lesion Count									
None, n (%)	1 (2.2)	5 (8.9)	0 (0.0)	3 (100.0)	0 (0.0)	2 (16.7)	7 (22.6)	0 (0.0)	1 (12.5)
Single site, n (%)	23 (50.0)	31 (55.4)	9 (56.3)	0 (0.0)	110 (69.6)	3 (25.0)	15 (48.4)	53 (72.6)	3 (37.5)
Multiple sites, n (%)	22 (47.8)	20 (35.7)	7 (43.8)	0 (0.0)	48 (30.4)	7 (58.3)	9 (29.0)	20 (27.4)	4 (50.0)
Lesion Location									
No lesion present	1 (2.2)	5 (8.9)	0 (0.0)	3 (100.0)	0 (0.0)	2 (16.7)	7 (22.6)	0 (0.0)	1 (12.5)
Head Lesion(s) Only	6 (13.0)	12 (21.4)	3 (18.8)	0 (0.0)	29 (18.4)	1 (8.3)	10 (32.3)	12 (16.4)	1 (12.5)
Thorax Lesion(s) Only	22 (47.8)	17 (30.4)	4 (25.0)	0 (0.0)	78 (49.4)	1 (8.3)	8 (25.8)	35 (48.0)	5 (62.5)
Body Lesion(s) Only	7 (15.2)	3 (5.4)	4 (25.0)	0 (0.0)	5 (3.2)	2 (16.7)	1 (3.2)	3 (4.1)	0 (0.0)
Head and Thorax Lesions	4 (8.7)	10 (17.9)	3 (18.8)	0 (0.0)	29 (18.4)	1 (8.3)	5 (16.1)	10 (13.7)	1 (12.5)
Head and Body Lesions	2 (4.4)	3 (5.4)	0 (0.0)	0 (0.0)	7 (4.4)	1 (8.3)	0 (0.0)	3 (4.1)	0 (0.0)
Thorax and Body Lesions	4 (8.7)	3 (5.4)	2 (12.5)	0 (0.0)	7 (4.4)	2 (16.7)	0 (0.0)	4 (5.5)	0 (0.0)
Head, Thorax, and Body Lesions	0 (0.0)	3 (5.4)	0 (0.0)	0 (0.0)	3 (1.9)	2 (16.7)	0 (0.0)	6 (8.2)	0 (0.0)

* Age is missing for two animals in B.1, six animals in B.2, one animal from D.3, 20 infected animals in D.2, and one animal in D.3.

**Table 5:** Antemortem skin-test results * for bovine tuberculosis-infected cattle by production type and herd.

Test Type	Caudal Fold Tuberculin (CFT) Test * (n = 376)			Comparative Cervical Tuberculin (CCT) Test * (n = 275)			
Test Result	Negative	Suspect	*p*-value	Negative	Suspect	Reactor	*p*-value ‡
All infected animals, n = 403	34 (9.0)	342 (91.0)		105 (38.2)	84 (30.6)	86 (31.3)	
Production Type			0.510				0.003
Beef, n = 118	7 (7.4)	88 (92.6)		8 (18.2)	12 (27.3)	24 (54.5)	
Dairy, n = 285	27 (9.6)	254 (90.4)		97 (42.0)	72 (31.2)	62 (26.8)	
Herd							
B.1, n = 46	6 (14.3)	36 (85.7)		8 (21.0)	6 (15.8)	24 (63.2)	
B.2, n = 56	0(0.0)	46 (100.0)		NP	NP	NP	
B.3 n = 16	1 (14.3)	6 (85.7)		0 (0.0)	0 (0.0)	6 (100.0)	
D.1, n = 3	0 (0.0)	3 (100.0)		NP	NP	NP	
D.2, n = 158	22 (14.1)	134 (85.9)		67 (58.8)	35 (30.7)	12 (10.5)	
D.3, n = 12	2 (18.2)	9 (81.8)		3 (33.3)	4 (44.5)	2 (22.2)	
D.4, n = 31	2 (6.7)	28 (93.3)		5 (17.8)	12 (42.9)	11 (39.3)	
D.5, n = 73	1 (1.4)	72 (98.6)		19 (26.0)	18 (24.7)	36 (49.3)	
D.6, n = 8	0 (0.0)	8 (100.0)		3 (42.9)	3 (42.9)	1 (14.2)	

* Antemortem test results included are those most proximate to the infected animal’s slaughter/necropsy. ‡ *p*-values for CCT results were calculated based on dichotomous results where CCT(N) = negative and CCT(S) or CCT(R) = positive due to low counts and herd removal practices. NP = not performed.

**Table 6:** Antemortem skin-test results for bovine tuberculosis-infected cattle by lesion count and location. * Antemortem test results included are those most proximate to the infected animal’s slaughter/necropsy. ‡ *p*-values for CCT results were calculated based on dichotomous results where CCT(N) = negative and CCT(S) or CCT(R) = positive due to low counts and herd removal practices.

Test Type	Caudal Fold Tuberculin (CFT) Test * (n = 376)			Comparative Cervical Tuberculin (CCT) Test * (n = 275)			
Test Result	Negative	Suspect	*p*-value	Negative	Suspect	Reactor	*p*-value ‡
Lesion Count			0.929				0.188
None, n (%)	2 (10.5)	17 (89.5)		2 (22.2)	4 (44.4)	3 (33.3)	
Single site, n (%)	20 (8.6)	212 (91.4)		75 (41.9)	57 (31.8)	47 (26.3)	
Multiple sites, n (%)	12 (9.6)	113 (90.4)		28 (32.2)	23 (26.4)	36 (41.4)	
Lesion Location							
No lesion present	2 (10.5)	17 (89.5)	0.686	2 (22.2)	4 (44.5)	3 (33.3)	0.490
Head Lesion(s) Only	6 (8.7)	63 (91.3)	1.00	20 (40.0)	20 (40.0)	10 (20.0)	0.872
Thorax Lesion(s) Only	11 (6.8)	151 (93.2)	0.207	49 (38.6)	47 (29.1)	41 (32.3)	0.902
Body Lesion(s) Only	5 (14.7)	18 (78.3)	0.046	6 (40.0)	4 (26.7)	5 (33.3)	1.00
Head and Thorax Lesions	6 (10.0)	54 (90.0)	0.806	18 (42.9)	8 (19.0)	16 (38.1)	0.496
Head and Body Lesions	2 (14.3)	12 (85.7)	0.367	3 (30.0)	2 (20.0)	5 (50.0)	0.746
Thorax and Body Lesions	1 (5.6)	17 (94.4)	1.00	2 (16.7)	4 (33.3)	6 (50.0)	0.140
Head, Thorax, and Body Lesions	1 (9.1)	10 (90.9)	1.00	5 (50.0)	5 (50.0)	0(0.0)	01.53

## Data Availability

Data were collected during official USDA investigations and are unavailable due to privacy restrictions.

## Abbreviations and Acronyms

The following abbreviations and acronyms are used in this manuscript:

bTB	Bovine tuberculosis
USDA	United States Department of Agriculture
STRAND	Searchable Test Results Application for NVSL Diagnostics
NVSL	National Veterinary Services Laboratories
Ln	Lymph node(s)

## Appendix A

**Table A1:** Region Description.

Region	Tissues Included
Head	medial retropharyngeal ln., lateral retropharyngeal ln., parotid ln., mandibular ln.
Thorax	mediastinal ln., tracheobronchial ln., lungs, heart
Body	mesenteric ln., hepatic ln., mammary ln., prescapular ln., popliteal ln., axillary ln., inguinal ln., liver
